# Multi-Physical Models of Bending Characteristics on the Double-Clamped Beam Switch for Flexible Electronic Devices Application

**DOI:** 10.3390/s20247074

**Published:** 2020-12-10

**Authors:** Lei Han, Lijun Chen, Ruijie Qin, Kang Wang, Zhiqiang Zhang, Meng Nie, Xiaodong Huang

**Affiliations:** Key Laboratory of MEMS of the Ministry of Education, Southeast University, Nanjing 210096, China; 220191411@seu.edu.cn (L.C.); 220171369@seu.edu.cn (R.Q.); 230188106@seu.edu.cn (K.W.); zqzhang@seu.edu.cn (Z.Z.); m_nie@seu.edu.cn (M.N.); xdhuang@seu.edu.cn (X.H.)

**Keywords:** bending characteristics, multi-physical models, double-clamped beam, flexible, MEMS switch

## Abstract

In this paper, multi-physical models of bending characteristics, including the static, dynamic and microwave models, are firstly proposed for the double-clamped beam switch based on flexible substrate. Both simulated and experimental verification have been carried out to prove that the changing regularity of the driving voltage and time of the switch is inversely proportional with the increase in the bending curvature of the flexible substrate. The microwave performance of the switch at the ON state is found to get worse with the increase in the bending curvature. The measured results indicate that when the bending curvature increases from 0 m^−1^ to 28.6 m^−1^, the measured driving voltage decreases from 90.0 V to 72.6 V with the error of 5.9% compared with the calculated results. The measured driving time decreases from 52.4 μs to 35.6 μs with the error of 16.7% compared with the calculated results. When the substrate bending curvature increases from 0 m^−1^ to 28.6 m^−1^, the measured reflection loss S_11_ of the switch gradually deteriorates from −27.1 dB to −22.0 dB with the error of 1.3 dB corresponding to the calculated results at 10 GHz. All the simulated and experimental results are consistent with the theoretical calculated results.

## 1. Introduction

The flexible electronic devices that manufacture electronic devices of organic or inorganic materials on a stretchable or bendable substrate are widely used in numerous fields including flexible communications [1,2,3,4], flexible sensors [5,6,7,8], flexible energy conversion [9,10], due to their advantages of miniaturization, bendability, high ductility, and low manufacturing cost. Flexible RF MEMS (Radio Frequency Micro-Electro-Mechanical Systems) devices [11,12,13,14,15,16] play an irreplaceable role in flexible communications and sensors for the excellent microwave performance and ultralow-power consumption. In particular, the flexible double-clamped beam switch, as an indispensable part of flexible RF MEMS devices, has become a research hotspot in recent years. As early as 2004, Wang et al. proposed a double-clamped beam switch based on flexible liquid crystal polymer (LCP) substrate with the working frequency of up to 40 GHz, with a lower loss appearing than the switch on silicon substrate [17]. In 2006, Zhang et al. first proposed a double-clamped beam switch fabricated on a flexible organic substrate (FR-4) by the wafer transfer technology (WTT), demonstrating a low insertion loss and high isolation [18]. In 2007, Patil et al. proposed a thin film silicon bridge micro-resonator on flexible polyethylene terephthalate substrates with a low processing temperature [19]. In 2020, Tiago et al. presented a double-clamped beam resonator based on 10 μm thick flexible polyimide substrate, exhibiting high quality factors and wide range of natural resonance frequencies [20]. However, these presented research studies cannot describe the behavior and performance variation of the flexible double-clamped beam switch under bending conditions for the deformation of both the substrate and beam, so multi-physical models of bending characteristics on the double-clamped beam switch are required. The modeling of the double-clamped beam RF MEMS switch based on traditional rigid substrate has been systematic and detailed. In 2014, Hatem et al. built a mathematical model for the MEMS switch, composed of two double-clamped beams and examined the transient behavior of this switch [21]. In 2015, Persano et al. estimated the pull-in voltage and the air gap between the bridge and the actuator of the GaAs-based double-clamped RF MEMS switch [22]. In 2018, Pertin et al. studied the pull-in voltage and microwave characteristics of the double-clamped beam switch, which was modeled with optimal geometry for a low pull-in voltage [23]. Nevertheless, because of the simultaneous deformation of the beam and flexible substrate under bending conditions, the multi-physical modeling of the double-clamped beam RF MEMS switch based on flexible substrate is full of challenges. At present, the research on the modeling of flexible double-clamped beam RF MEMS switch under bending conditions is only limited to the static mechanical modeling, and other aspects of modeling research are still lacking. In 2016, Han et al. studied the effects of the double-clamped beam RF MEMS switch based on flexible liquid crystal polymer (LCP) substrates under bending conditions on the static mechanical property of the switch [24], but only the driving voltage of the switch was introduced in this study. In order to study the bending characteristics of the double-clamped beam switch comprehensively, multi-physical models composed of the static, dynamic and microwave models need to be established.

In this paper, systematic multi-physical models of the flexible spring-like double-clamped beam RF MEMS switch under bending conditions, including the static, dynamic and microwave models, are proposed for the first time. The spring-like double-clamped beam switch is designed and fabricated. The calculation, measurement and simulation of the bending characteristics on the actual spring-like double-clamped beam switch are carried out to validate the models. Results of this study indicate that the driving voltage and time of the switch decrease with the increase in the bending curvature. Additionally, the microwave performance of the switch is found to get worse following the increase in the bending curvature. Both simulated and experimental results are in good agreement with the theoretical calculated results.

## 2. Modeling

**Figure 1:**
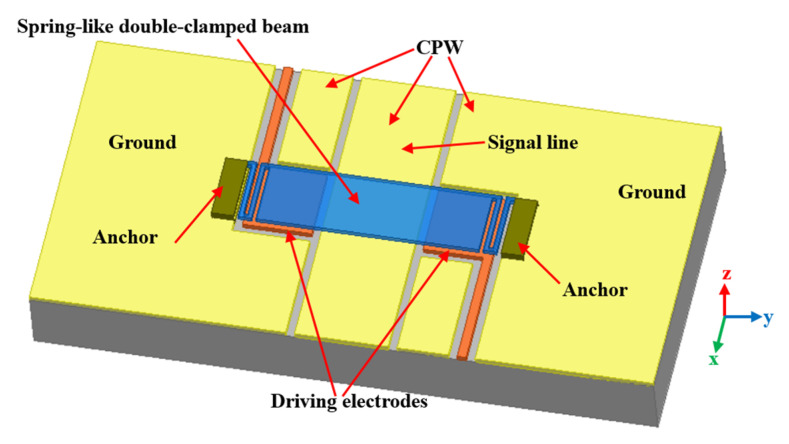
3-D structure diagram of the double-clamped beam switch.

The structure of the double-clamped beam switch is shown in Figure 1, the spring-like double-clamped beam is suspended on the signal line of coplanar waveguide (CPW), and two driving electrodes are located at two sides of the signal line underneath the double-clamped beam. When voltage is applied on the driving electrodes, electrostatic force comes into being between the beam and the electrodes to pull down the beam. When the double-clamped beam touches the signal line, the switch turns into the OFF state.

### 2.1. Static Model of Bending Characteristics

The driving voltage of the double-clamped beam switch under the flat condition, which considers both the initial z-direction elastic constant and the additional elastic constant caused by stress, can be obtained by [24]

(1) $$V=\frac{2g}{3}\sqrt{\frac{kg}{3\varepsilon_{r}\varepsilon_{0}A}},$$

where g is the gap between the beam and driving electrodes, A is the overlapping area between the double-clamped beam and the driving electrodes, $k=k^{\prime }+k^{\prime \prime }$ is the equivalent elastic constant of the spring-like double-clamped beam, in which $k^{\prime }$ means the z-direction elastic constant of the beam, $k^{\prime \prime }$ means the additional elastic constant caused by stress. The two elastic constants can be obtained from the following derivation.

**Figure 2:**
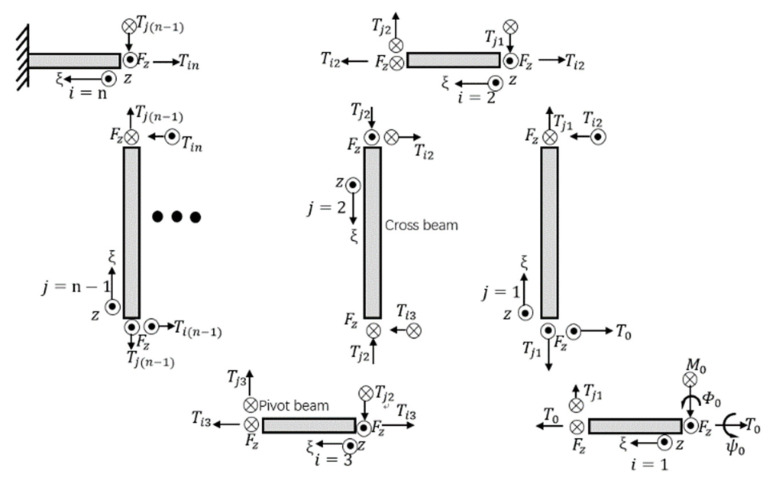
The free body diagram of the spring structure.

The free body diagram of the spring structure of the beam with the vertical force $F_{z}$ applied is shown in Figure 2, where $T_{mn}$ is the torsion for the *n*th pivot beam (when *m* = *i*) or *n*th cross beam (when *m* = *j*) and $\phi_{0}$, $\psi_{0}$ represent the rotation angles, which are enslaved to the guided-end condition including the external bending moment $M_{0}$. ξ is the distance from the guided-end in the direction along the length of each beam. According to the energy method, the z-direction elastic constant of the spring-like double-clamped beam can be calculated as following, where *n* represents the number of the pivot beam. When *n* is even

(2) $$k_{z}=\frac{12S_{ea}S_{eb}S_{ga}S_{gb}}{\left\{\begin{array}{l} S_{eb}S_{ga}a^{2}(S_{gb}a+S_{eb}b)n^{3}-3S_{ea}S_{eb}S_{ga}a^{2}bn^{2}+\\ S_{ea}b(2S_{eb}S_{ga}a^{2}+3S_{eb}S_{gb}ab+S_{ga}S_{gb}b^{2})n-\\ S_{ea}S_{ga}S_{gb}b^{3} \end{array}\right\}},$$

and when *n* is odd

(3) $$k_{z}=\frac{12S_{ea}S_{eb}S_{ga}S_{gb}(S_{ga}b(n-1)+S_{eb}an)}{\left\{\begin{array}{l} (S_{eb}S_{ga}a^{2}(S_{eb}S_{gb}a^{2}+(S_{ea}S_{eb}+S_{ga}S_{gb})ab+\\ S_{ea}S_{ga}b^{2})n^{4}-S_{eb}S_{ga}a^{2}b((3S_{ea}S_{eb}+\\ S_{ga}S_{gb})a+4S_{ea}S_{ga}b)n^{3}+S_{ea}b(2S_{eb}^{2}S_{ga}a^{3}+\\ (5S_{eb}S_{ga}^{2}+3S_{eb}^{2}S_{gb})a^{2}b+4S_{eb}S_{ga}ab^{2}+\\ S_{ga}^{2}S_{gb}b^{3})n^{2}-2S_{ea}S_{ga}b^{2}(S_{eb}S_{ga}a^{2}+2S_{eb}S_{ga}ab+\\ S_{ga}S_{gb}b^{2})n+S_{ea}S_{gb}b^{2}(S_{ga}^{2}b^{2}-3S_{eb}^{2}a^{2})) \end{array}\right\}},$$

where a and b are the lengths of the pivot beam and cross beam, respectively. $S_{ey}=EI_{x,y}$, $S_{gy}=GJ_{y}$, in which $I_{x,y}$ represents the moment of inertia for the pivot beam (when *y* = *a*) and cross beam (when *y* = *b*) for the x-direction, $J_{y}$ represents the torsion constant of the pivot beam and cross beam.

As the spring-like double-clamped beam we employ is composed of three pivot beams and two cross beams as shown in Figure 1, the z-direction elastic constant of the spring-like double-clamped beam is $k^{\prime }=2k_{z}$.

If there is a distributed force q(x), an additional elastic constant caused by stress is induced in the vertical direction of the beam, which can be calculated by

(4) $$k^{\prime \prime }=\frac{P}{Y^{\prime \prime }}=\frac{2\int_{x_{1}}^{x_{1}+a}q(z)dz}{Y^{\prime \prime }}=\frac{\int_{x_{1}}^{x_{1}+a}q(z)dz}{\int_{x_{1}}^{x_{1}+a}\frac{q(z)}{2S}(l-z)dz},$$

where S=σtw(1−ν), σ is the stress, t, w, v are the thickness, width and Poisson ratio of the spring-like double-clamped beam, respectively. 

However, when the switch is bending, the equivalent elastic constant of the spring-like double-clamped beam as well as the gap between the beam and driving electrodes will be changed, leading to the variation of the driving voltage.

**Figure 3:**
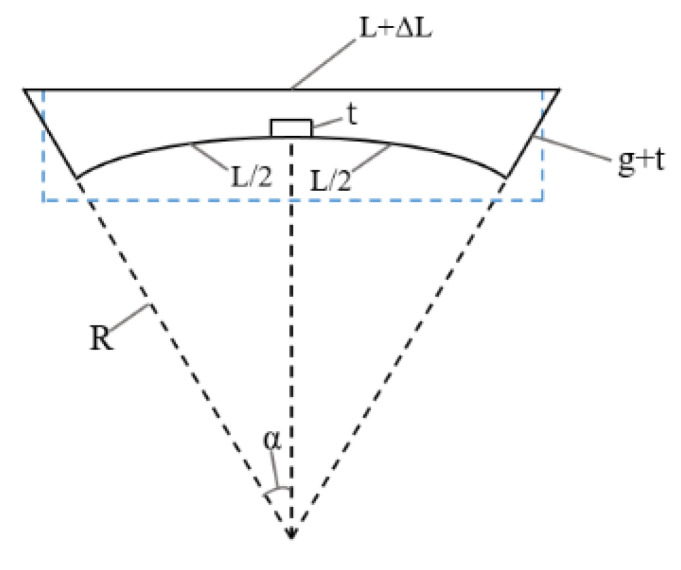
2-D model of the double-clamped beam under the bending condition.

For an explicit explanation, a 2-D model of the double-clamped beam under the bending condition is shown in Figure 3. On the one hand, when the substrate is bending, the tensile stress is induced into the beam, leading to the elongation ΔL of the beam and the force caused by the elongation of the beam can be calculated by $S^{\prime }=\sigma_{0}tw=\Delta L{k^{\prime }}_{y}$, where ${k^{\prime }}_{y}$ is the revised y-direction elastic constant of the beam. The additional elastic constant of the spring-like double-clamped beam under bending conditions can be calculated by substituting the revised $S^{\prime }$ into Equation (4). On the other hand, when the substrate is bending, the gap between the spring-like double-clamped beam and driving electrodes is decreased and the revised gap can be calculated by $g^{\prime }(a)=(R+g+t)\cos(\frac{90L}{\pi R})-(R+t)\cos(\frac{90a}{\pi R})$, where t is the thickness of the signal line, a is the distance relative to the center of the signal line.

In conclusion, we can calculate the driving voltage of the spring-like double-clamped beam switch under different bending conditions, by substituting the revised gap $g^{\prime }$ and equivalent elastic constant k into Equation (1).

### 2.2. Dynamic Model of Bending Characteristics

The dynamic equation of the RF MEMS double-clamped beam switch is given by the d’Alembert’s formula [25]

(5) $$m\frac{d^{2}z}{dt^{2}}+b\frac{dz}{dt}+kz+k_{s}z^{3}=F_{e},$$

where m is the effective mass of the double-clamped beam, $b=3\mu {\left(wl\right)}^{2}/(2\pi g^{3})$ is the damping constant of the double-clamped beam, in which g is the initial gap between the beam and the driving electrodes, μ is the viscosity coefficient of the ideal gas, z is the vertical displacement of the beam, k is the effective elastic constant of the beam and $k_{s}=\pi^{4}Ewt/(8l^{3})$ is stress-stiffening elastic constant. $F_{e}=\varepsilon_{r}\varepsilon_{0}AV^{2}/\left[2{\left(g-z\right)}^{2}\right]$ is electrostatic force between the beam and the driving electrodes, in which A is the overlapping area between the beam and the driving electrodes, V is the voltage applied to the driving electrodes.

As mentioned in Section 2.1., the initial gap between the beam and the driving electrodes as well as the effective elastic constant of the beam under different bending conditions have been deduced, leading to the revised b, k and $F_{e}$ under bending conditions. Substitute these parameters into Equation (5) and we can obtain the dynamic equation of the spring-like double-clamped beam switch under bending conditions

(6) $$m\frac{d^{2}z}{dt^{2}}+\frac{3\mu {\left(wl\right)}^{2}}{2\pi {g^{\prime }}^{3}}\frac{dz}{dt}+(k^{\prime }+k^{\prime \prime })z+k_{s}z^{3}=\frac{\varepsilon_{r}\varepsilon_{0}AV^{2}}{2{\left[g^{\prime }-z\right]}^{2}},$$

where $g^{\prime }$ is the revised gap between the double-clamped beam and the driving electrodes under bending conditions. By solving the dynamic equation (Equation (6)), the driving time under different bending conditions is deduced.

### 2.3. Microwave Model of Bending Characteristics

When the RF MEMS switch is at the ON state with the flat substrate, without regard to the resistance, conductor loss and dielectric loss of the double-clamped beam, the reflection loss *S*_11_ can be calculated by

(7) $$S_{11}=\frac{-j\omega C_{u}Z_{0}}{2(1-\omega^{2}L_{t}C_{u})+j\omega C_{u}Z_{0}},$$

where $Z_{0}$ is the characteristic impedance of the CPW transmission line, $C_{u}$ is the capacitance between the double-clamped beam and the signal line of the CPW transmission line at the ON state of the switch, $L_{t}$ is the total inductance of the spring-like double-clamped beam, which is equal to the sum of the spring-like inductance and the flat-film inductance. The spring-like inductance can be expressed by [26]

(8) $$L_{s}=0.00266\times a^{0.0603}\times b^{0.4429}\times N^{0.954}\times d^{0.606}\times w_{s}^{-0.173},$$

where N is the number of cross beams, a, b are the length of the cross beam and pivot beam closed to the anchors, d is the gap between the cross beam and $w_{s}$ is width of the spring-like inductance.

The flat-film inductance can be calculated by [26]

(9) $$L_{f}=0.002D\times \left(\ln\frac{2D}{W}+0.50049+\frac{W}{3D}\right),$$

where D and W are the length and width of the flat-film inductance, respectively.

According to the analysis in Section 2.1., when the flexible substrate is bending, the gap between the CPW signal line and the double-clamped beam decreases, which increases the ON state capacitance $C_{u}$. When we calculate the ON state capacitance $C_{u}$ of the double-clamped beam switch under bending conditions, the influence of the edge capacitance also needs to be considered. Then, the calculated formula for the revised ON state capacitance $C_{u}$ of the switch under bending conditions is deduced by

(10) $$C_{u}=K_{c}C_{pp}=K_{c}\int_{\frac{L-W}{2}}^{\frac{L+W}{2}}\frac{\varepsilon_{0}\varepsilon_{r}wdx}{(R+g)\cos(\frac{90L}{\pi R})-\sqrt{R^{2}-{\left(x-\frac{L}{2}\right)}^{2}}},$$

where $C_{pp}$ is the parallel plate capacitance, $K_{c}$ is the ratio of edge capacitance, which can be obtained by [27]

(11) $$K_{c}=\left[1+g(1+\ln(2\pi w/g))/(\pi w)\right]\;\left[1+g(1+\ln(2\pi W/g))/(\pi W)\right],$$

According to Equation (11), we can find that the ratio $K_{c}$ of edge capacitance decreases with the decline of the gap g between the beam and the signal line under bending conditions. This can be explained by the reason that when the gap between the beam and the signal line decreases with the increase in bending curvature, the parallel plate capacitance accounts for a greater proportion in ON state capacitance $C_{u}$, corresponding to the decrease in the ratio of the edge capacitance.

By substituting the revised ON state capacitance $C_{u}$ into Equation (7), the reflection loss *S*_11_ of the double-clamped beam switch under different bending conditions can be calculated.

## 3. Design and Fabrication

**Figure 4:**
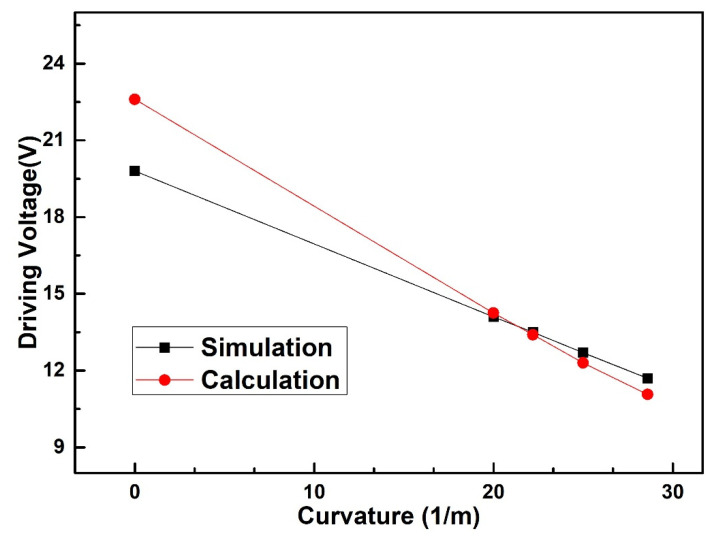
Simulated driving voltage of the spring-like double-clamped beam switch with different substrate curvatures.

With the simulation of ANSYS software, the length and width of the spring-like double-clamped beam structure are designed to be 600 μm and 100 μm. Au is used as the material for the double-clamped beam switch with LCP (Liquid Crystal Polymer) as the flexible substrate. If Au is replaced by other materials, the driving voltage of the switch will change, which depends on the characteristics of the selected alternative materials such as Poisson ratio, density and so on. The material characteristics and geometric parameters of the spring-like double-clamped beam switch are listed in Table 1 and Table 2. The simulated driving voltage of the spring-like double-clamped beam switch under different bending curvatures is shown in Figure 4.

According to the simulated results of Figure 4, we can find that the simulated driving voltage of the spring-like double-clamped beam switch decreases from 19.8 V to 11.7 V as the bending curvature of the substrate increases. The rate of simulated driving voltage change is 40.9% when the bending curvature increases from 0 m^−1^ to 28.6 m^−1^. Meanwhile, the error between the simulated results and calculated results is within 14.1%, regarding the simulated results as the truth value. This error can be explained by the reason that the calculated model we established is a two-dimensional plane model, which does not take the width of the spring-like double-clamped beam and the two edges along the width direction of the spring-like double-clamped beam into account. Correspondingly, the fringe field, which can bring an additional electrostatic force, is ignored, leading to an error in the calculated driving voltage. In addition, the simulated value is increasingly close to the calculated value with the increase in the bending curvature, which can be explained by the smaller impact of fringe capacitance and additional electrostatic force caused by the approach of the beam and the driving electrode with the increase in the bending curvature. 

**Figure 5:**
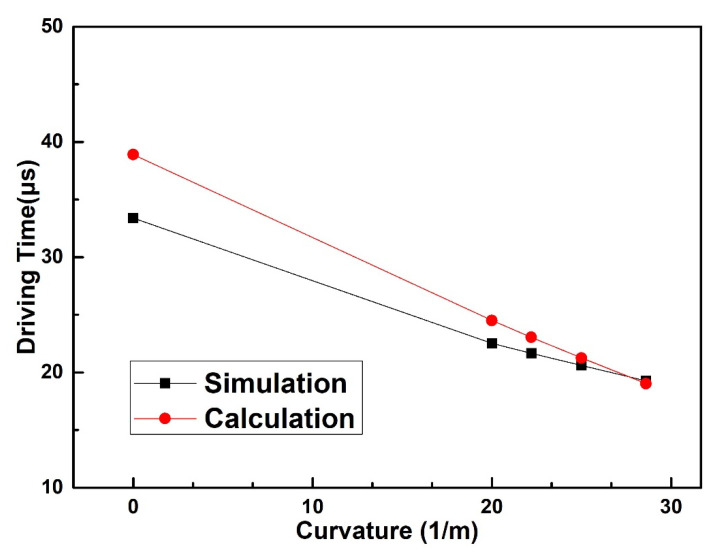
Simulated driving time of the spring-like double-clamped beam switch with different substrate curvatures.

The driving time of the spring-like double-clamped beam switch is also simulated by ANSYS with the same applied voltage of 25 V, and the simulated results are shown in Figure 5.

From the results shown in Figure 5, it is evident that the simulated driving time of the spring-like double-clamped beam switch decreases from 33.4 μs to 19.3 μs with the increase in the bending curvature, and the rate of simulated driving time change is 42.3% when the bending curvature increases from 0 m^−1^ to 28.6 m^−1^. This can be explained by the reason that with the increase in the bending curvature, the gap between the spring-like double-clamped beam and driving electrodes decreases, leading to the increase in electrostatic force between the spring-like double-clamped beam and driving electrodes with the same voltage applied. As a result, the driving time decreases with the increase in the bending curvature. The maximum error between the simulated results and calculated results of the driving time is within 16.5%, with the simulated results as the truth value, which is mainly caused by the reason that the calculated model we established is a two-dimensional plane model, which does not consider the width of the spring-like double-clamped beam and the two edges along the width direction of the spring-like double-clamped beam. Correspondingly, the fringe field, which can bring an additional electrostatic force is ignored, leading to a larger calculated driving voltage. As a result, the driving time we calculated is larger than the simulated driving time. It is also clear that the simulated value is increasingly close to the calculated value with the increase in the bending curvature. This can be explained by the reason that the approach of the beam and the signal line with the increase in the bending curvature leads to the smaller impact of fringe capacitance and additional electrostatic force.

**Figure 6:**
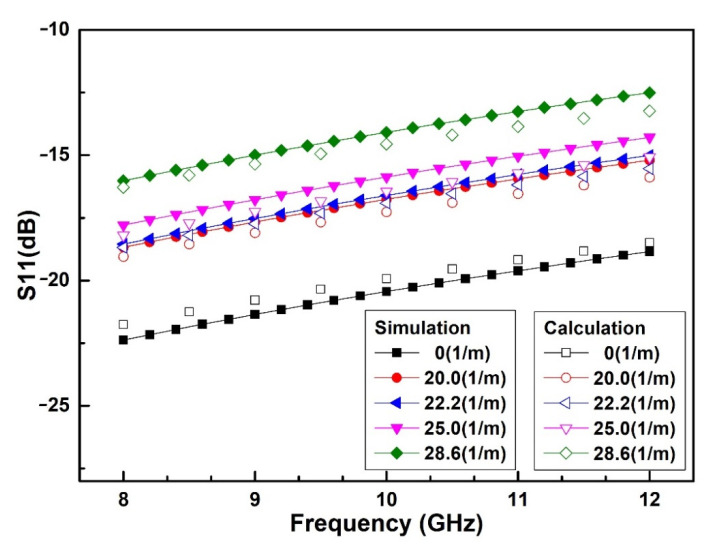
Simulated reflection loss S_11_ of the spring-like double-clamped beam switch with different substrate curvatures.

With the LCP flexible substrate selected with a thickness of 100 μm and dielectric constant of 3.0, the G/S/G (Ground/Signal/Ground) dimension of the CPW is designed to be 20 μm/210 μm/20 μm to achieve the characteristic impedance of 50 Ω. On this basis, HFSS software is used to simulate the microwave performance of the switch under different bending curvatures including 0 m^−1^, 20.0 m^−1^, 22.2 m^−1^, 25.0 m^−1^ and 28.6 m^−1^, respectively. The simulated and calculated results of microwave performance are shown in Figure 6.

As we can see from Figure 6, when the substrate bending curvature increases from 0 m^−1^ to 28.6 m^−1^, the simulated reflection loss S_11_ of the switch at the ON state gradually deteriorates from −20.4 dB to −14.1 dB at 10 GHz. The reason for the deterioration of microwave performance is that when the substrate is bent, the gap between the double-clamped beam and the signal line becomes smaller, causing the growth of the parallel capacitance of the ON state, which leads to the mismatch of the microwave performance. The changing regularity is completely consistent with the theoretical model, and the simulated results of S_11_ differ from the calculated results by less than 0.8 dB in the frequency range of 8–12 GHz. The cause of the error of S_11_ can be explained by the reason that the characteristic impedance of CPW in the simulation is not a precise 50 Ω, while the characteristic impedance is considered to be 50 Ω in the microwave model.

**Figure 7:**
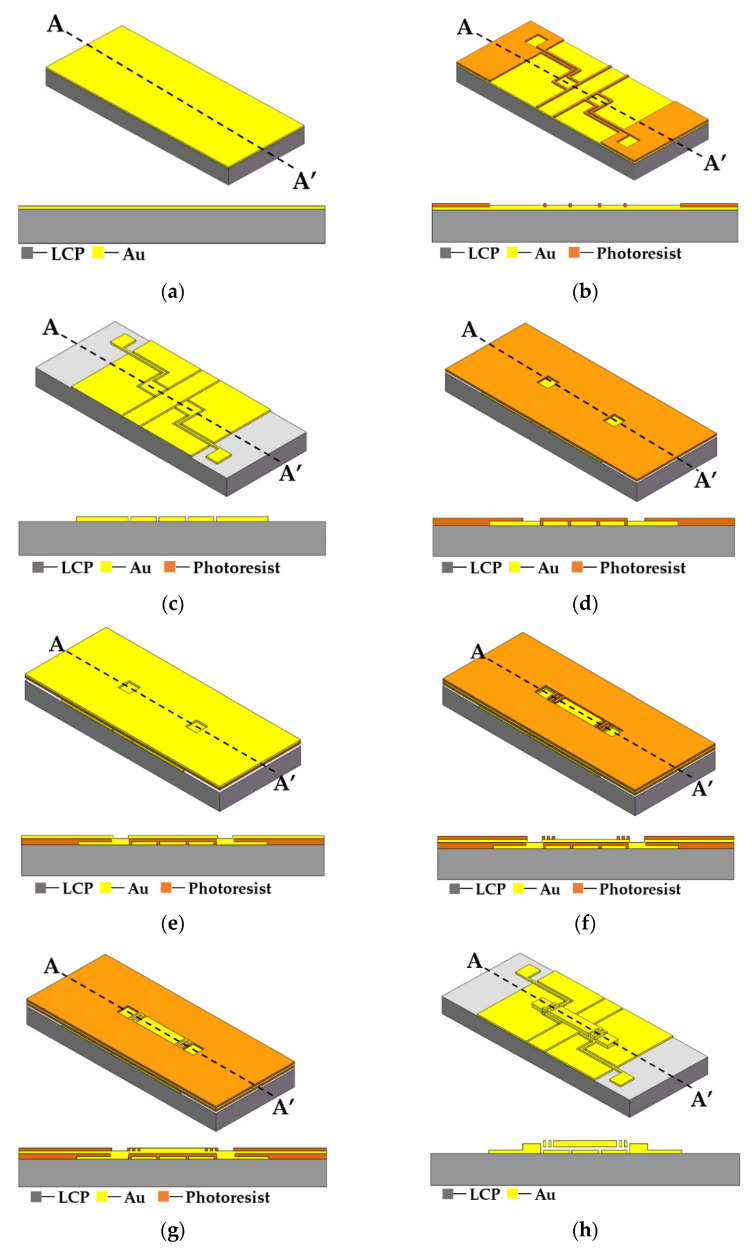

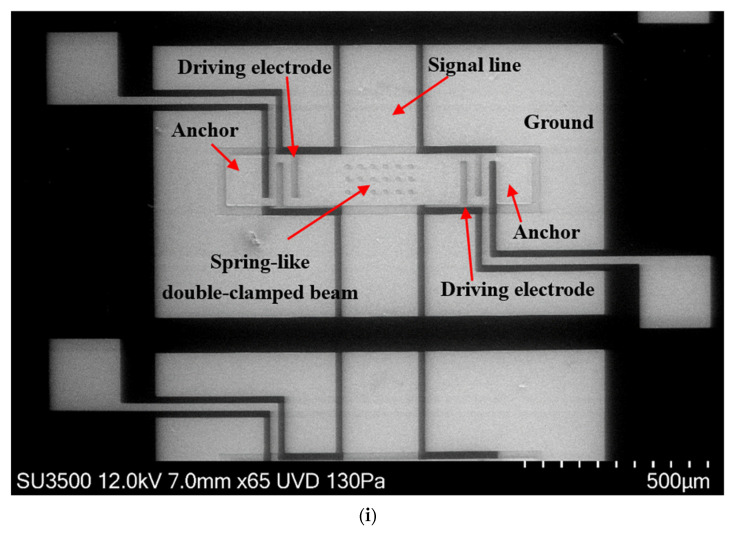
Process steps and SEM image of the double-clamped beam switch. (**a**) Sputtering Au seed layer. (**b**) Patterning and electroplating Au. (**c**) Etching redundant seed layer to form the transmission line and driving electrodes. (**d**) Patterning photoresist. (**e**) Sputtering a seed layer to form the anchors. (**f**) Patterning photoresist. (**g**) Electroplating Au to form the double-clamped beam. (**h**) Removing the redundant seed layer and photoresist. (**i**) The SEM image of the spring-like double-clamped beam switch.

The RF MEMS spring-like double-clamped beam switch is fabricated on LCP flexible substrate, as shown in Figure 7. Firstly, a Rogers single-sided copper-clad LCP substrate with a thickness of 100 μm is prepared and Au is sputtered as the seed layer, as shown in Figure 7a. Then, Au is patterned and electroplated, and the redundant seed layer is etched to form the transmission line and driving electrodes, as shown in Figure 7b,c. Next, the photoresist is patterned again to form the anchors of the double-clamped beam and the seed layer is sputtered, as shown in Figure 7d,e. After that, the photoresist is patterned, and Au is electroplated to develop the double-clamped beam, as shown in Figure 7f,g. Finally, the seed layer and photoresist are removed to release the structure, as shown in Figure 7h and the scanning electron microscopy (SEM) image of the fabricated switch is shown in Figure 7i.

## 4. Experimental Results

**Figure 8:**
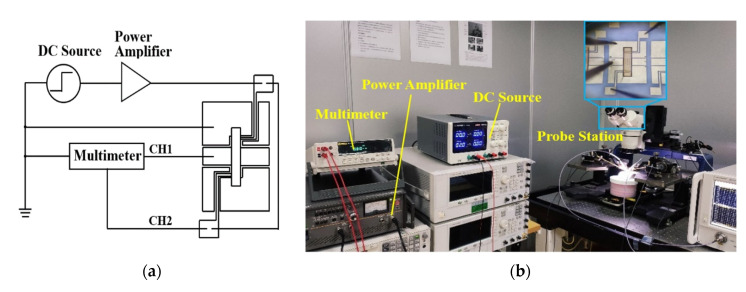
(**a**) Test circuit diagram for the driving voltage. (**b**) Test platform for the driving voltage.

**Figure 9:**
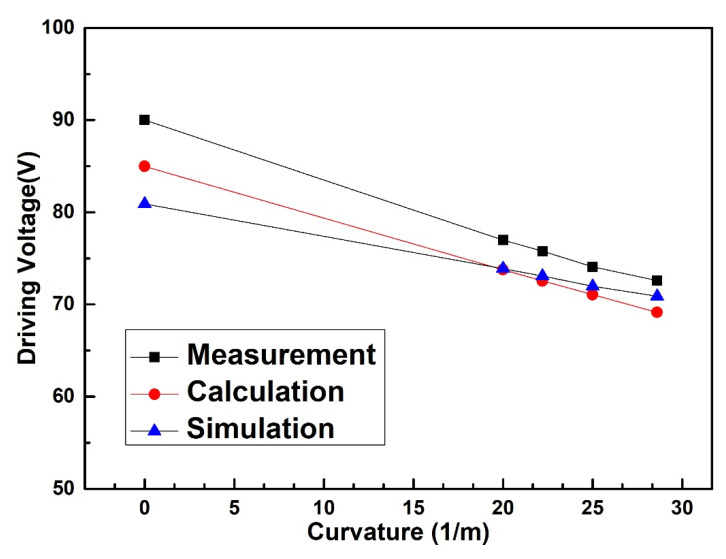
Measured driving voltage of the spring-like double-clamped beam switch with different bending curvatures.

The driving voltage of the manufactured spring-like double-clamped beam switch under bending conditions has been tested using various instruments including a power amplifier, a DC source, a multimeter and an Agilent N5244A PNA-X network analyzer with 150 μm-pitch Cascade Micro-Tech G/S/G CPW probe station as shown in Figure 8. The actual size of the fabricated switch profile was measured by the NANOVEA CRS-25XY 3-D profile meter and is shown in Table 3. According to the fabrication process steps shown in Figure 7, the thickness of the beam of 2.4 μm is measured indirectly by subtracting the altitude of the signal line of 2.6 μm from the altitude of the anchors of 5.0 μm. The measurement uncertainties of the thickness and gap are ±0.1 μm and the measurement uncertainties of width and length are ±0.5 μm, correspondingly. The experimental results, simulated results, and the theoretically calculated results of the driving voltage of the switch under different bending conditions are shown in Figure 9.

According to the experimental results in Figure 9, the driving voltage of the flexible double-clamped beam switch decreases with the increase in bending curvature. When the bending curvature of the flexible substrate varies from 0 m^−1^ to 28.6 m^−1^, the measured driving voltage of the double-clamped beam switch decreases from 90.0 V to 72.6 V. The rate of measured driving voltage change is 19.3% when the bending curvature increases from 0 m^−1^ to 28.6 m^−1^. The static model demonstrates that, under the bending conditions, the distance between the double-clamped beam and the driving electrode decreases, which finally decreases the driving voltage. The simulated results and measured results of the driving voltage agree well with the calculated results and the relationship between the simulated results and the calculated results shows the same trend as that shown in Figure 4. The maximum error between the simulated results and calculated results is within 4.1% when the simulated results are regarded as the truth value. Meanwhile, the error between the measured results and the theoretically calculated results is within 5.9% with the measured results as the truth value, indicating that the measured results agree well with the calculated value. The error between the measured results and the calculated results can be explained by the reason that the calculated model does not consider the width of the spring-like double-clamped beam and the two edges along the width direction of the spring-like double-clamped beam. Correspondingly, the fringe field, which can bring an additional electrostatic force, is ignored, leading to an error in the calculated driving voltage. In general, the static modeling we built can better reflect the variation of the driving voltage of the spring-like double-clamped beam switch under bending conditions.

**Figure 10:**
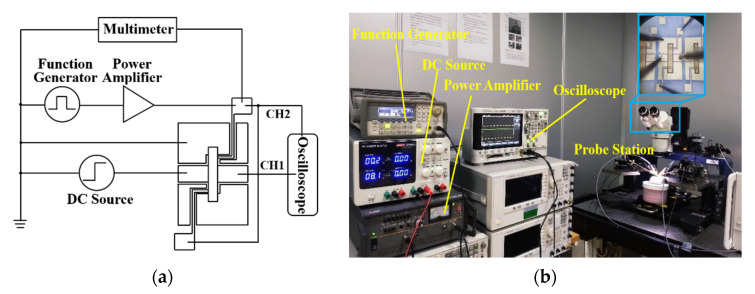
(**a**) Test circuit diagram for the driving time. (**b**) Test platform for the driving time.

The driving time of the flexible RF MEMS spring-like double-clamped beam switch under different bending curvatures is measured. An oscilloscope and a function generator are added, as shown in Figure 10.

**Figure 11:**
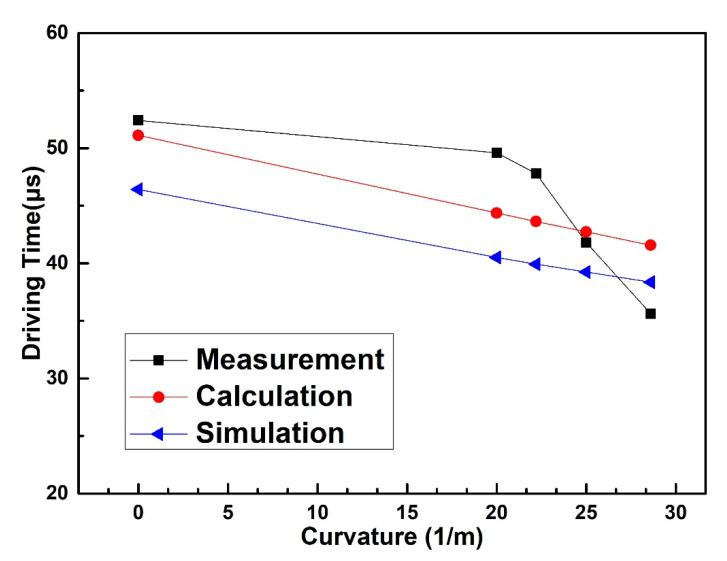
Measured driving time of the spring-like double-clamped beam switch with different substrate curvatures.

The measured results, simulated results and theoretically calculated results of the driving time of the spring-like double-clamped beam switch under different bending conditions are shown in Figure 11. During the measurement, the voltage applied to the driving electrodes remains unchanged with the value of 115 V, and only the bending curvature of the substrate varies. As can be seen from Figure 11, when the bending curvature of the flexible substrate increases from 0 m^−1^ to 28.6 m^−1^, the measured driving time of the spring-like double-clamped beam switch continuously decreases from 52.4 μs to 35.6 μs. The rate of measured driving time change is 32.1% when the bending curvature varies from 0 m^−1^ to 28.6 m^−1^. The dynamic model demonstrates that under the bending conditions, the driving voltage of the switch and the gap between the beam and driving electrodes decrease, which increases the driving force between the beam and driving electrodes and decreases the displacement of beam movement. As a result, the driving time declines with the increase in the bending curvature when the same voltage is applied. After analysis of the results, we can find that the error between the calculated results and simulated results is within 10.1% with the simulated results as the truth value. The simulated curve is almost parallel to the calculated curve, which can be explained by the reason that the large gap between the beam and the driving electrode results in small fringe capacitance. Correspondingly, the measured results of the driving time show the same trend as the theoretically calculated results with the maximum error of 16.7% regarding the measured results as the truth value. The error can be explained by the reason that the spring-like double-clamped beam warps upward due to the fabrication process limitation. As a result, when the bending curvature is small, the stress introduced into the beam caused by the bending of the substrate is too small to straighten the beam completely and the middle part of the beam remains buckled upward with little deformation, leading to the slow decline of the gap between the beam and the driving electrode. As the bending curvature increases, the stress introduced into the beam is large enough to straighten the middle part of the beam, leading to the approach of the beam to the driving electrode when the flexible substrate is continuously bent upward. As a result, the gap between the beam and the driving electrode decreases rapidly with the increase in the bending curvature. Therefore, the measured results of the gap between the beam and the driving electrodes decline more slowly than the calculated results when the bending curvature increases from 0 m^−1^ to 22.2 m^−1^ and more rapidly than the calculated results when the bending curvature increases from 22.2 m^−1^ to 28.6 m^−1^. Ultimately, the driving time we calculated is smaller than the measured driving time when the bending curvature increases from 0 m^−1^ to 22.2 m^−1^ and larger than the measured driving time when the bending curvature increases from 22.2 m^−1^ to 28.6 m^−1^. In general, the dynamic modeling proposed in this paper can reflect the variation of the driving time of the spring-like double-clamped beam switch under bending conditions well.

**Figure 12:**
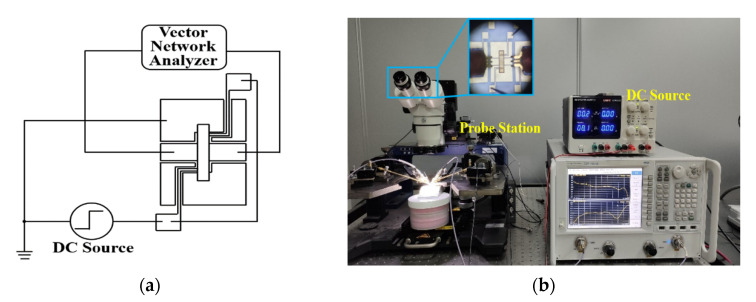
(**a**) Test circuit diagram for the microwave performance. (**b**) Test platform for the microwave performance.

**Figure 13:**
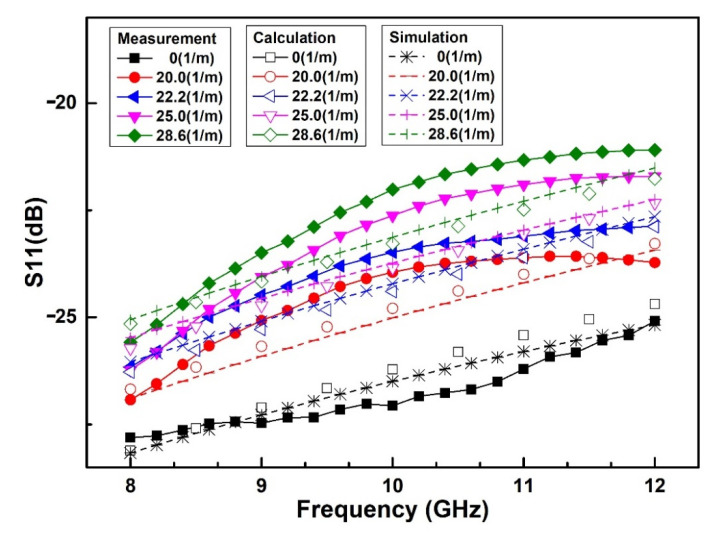
Measured reflection loss S_11_ of the spring-like double-clamped beam switch with different substrate curvatures.

Finally, the microwave performance of the spring-like double-clamped beam switch based on a flexible substrate is measured. The test circuit diagram for the microwave performance is shown in Figure 12a, and Figure 12b is the test platform including a DC source and an Agilent N5244A PNA-X network analyzer with a 150 μm-pitch Cascade Micro-Tech G/S/G CPW probe station. The microwave performance of the flexible RF MEMS spring-like double-clamped beam switch is tested at the curvature of 0 m^−1^, 20.0 m^−1^, 22.2 m^−1^, 25.0 m^−1^ and 28.6 m^−1^. The experimental results are shown in Figure 13.

As can be seen from Figure 13, when the substrate bending curvature increases from 0 m^−1^ to 28.6 m^−1^, the measured reflection loss S_11_ of switch at the ON state gradually deteriorates from −27.1 dB to −22.0 dB at 10 GHz. The reason for the degradation of the microwave performance of the spring-like double-clamped beam switch after bending is that when the substrate is bent, the gap between the beam and signal line becomes smaller, resulting in an increase in the parallel capacitance of the switch at the ON state, so the microwave performance of the switch at the ON state deteriorates. The measured results, simulated results, calculated results are well consistent with the others and the error between the calculated results and simulated results of S_11_ is within 0.5 dB. Correspondingly, the error between the measured results and calculated results of S_11_ is within 1.3 dB, which can be explained by the reason that the characteristic impedance of the CPW measurement is not a precise 50 Ω. In general, the microwave modeling proposed in this paper can reflect the variation of the reflection loss S_11_ of the spring-like double-clamped beam switch under bending conditions well.

## 5. Conclusions

This paper firstly proposes systematic multi-physical models of bending characteristics, including the static, dynamic and microwave models for the flexible spring-like double-clamped beam switch, which can quantitatively analyze the performance drift of the spring-like double-clamped beam switch under bending conditions. According to the multi-physical models, the spring-like double-clamped beam switches based on LCP flexible substrate are designed and fabricated. The experimental results prove that the driving voltage and time of the switch decreases with the increase in the bending curvature and the microwave performance of the switch at the ON state is found to deteriorate with the increase in the bending curvature. The measured results indicate that when the substrate bending curvature increases from 0 m^−1^ to 28.6 m^−1^, the measured driving voltage of the spring-like double-clamped beam switch decreases from 90.0 V to 72.6 V with the error within 5.9% corresponding to the calculated results. As the substrate bending curvature increases from 0 m^−1^ to 28.6 m^−1^, the measured driving time of the spring-like double-clamped beam switch decreases from 52.4 μs to 35.6 μs and the difference between the measured results and the calculated results of driving time is within 16.7%. When the substrate bending curvature increases from 0 m^−1^ to 28.6 m^−1^, the measured reflection loss S_11_ of the switch at the ON state gradually deteriorates from −27.1 dB to −22.0 dB with the error of 1.3 dB corresponding to the calculated results at 10 GHz. All the simulated and experimental results are in good agreement with the theoretical calculated results.

## Figures and Tables

**Table 1:** Material characteristics of the spring-like double-clamped beam switch.

Material	Au	LCP
Resistivity ρ (μΩ·cm)	2.35	N/A
Thermal conductivity κ (W/cm·K)	3.18	0.002
Thermal expansion coefficient α (×10^−6^/K)	14.2	17
Young’s modulus E (GPa)	78	2.225
Density δ (kg/m^3^)	19,300	1400
Specific heat capacity C (J/kg·K)	128	1340
Poisson’s ratio	0.42	0.49

**Table 2:** Geometric parameters of the spring-like double-clamped beam switch.

Item	Size (μm)
Width of the beam	100
Length of the beam	600
Thickness of the beam	2.5
Width of the driving electrode	100
Length of the driving electrode	155
Thickness of the driving electrode	2
Gap between the original beam and driving electrode	3

**Table 3:** Geometric parameters of the actual machining switch.

Item	Size (μm)
Width of the beam	140
Length of the beam	650
Thickness of the beam	2.4
Gap between the original beam and driving electrode	10.6
